# Complete Genome Sequence of Pediococcus pentosaceus Strain GDIAS 001

**DOI:** 10.1128/MRA.01586-19

**Published:** 2020-03-19

**Authors:** Maopeng Song, Yongfei Wang, Xianyong Ma, Dun Deng

**Affiliations:** a Department of Bioengineering, School of Life Sciences, Jinan University, Guangzhou, China; b State Key Laboratory of Animal Breeding, South China Key Laboratory of Animal Nutrition and Feed, Ministry of Agriculture, Institute of Animal Science, Guangdong Academy of Agricultural Sciences, Animal Breeding and Nutrition Public Laboratory, Poultry Meat Quality, Quality Control and Evaluation Engineering Technology Center, Guangzhou, China; University of Arizona

## Abstract

Pediococcus pentosaceus strain GDIAS 001 was isolated from a tapioca sample in Guangzou, China. The genome of GDIAS 001 was assembled using single-molecule real-time (SMRT) sequencing, and it contains 1 chromosome of 1.83 Mbp and 1,835 protein-coding genes, 71 RNA genes, and 56 tRNA genes.

## ANNOUNCEMENT

Pediococcus pentosaceus is a member of the lactic acid bacteria and facultative anaerobic Gram-positive bacteria (1). P. pentosaceus is usually found in food and feed materials. Those strains can improve food flavor (2) and taste (3), especially for meat and cheese (4). Some P. pentosaceus strains are also effective in resisting pathogenic bacteria (5, 6). Thus, P. pentosaceus has broad application prospects in the food, medicine, and feed industries.

Here, Pediococcus pentosaceus strain GDIAS 001 was isolated from tapioca. It was cultured on MRS agar plates (7) at 30°C. Six passages of GDIAS 001 were stored in 20% glycerol at −80°C. Then, the genomic DNA of GDIAS 001 was prepared and sequenced with the following steps. First, a single colony was cultured in MRS liquid culture. Second, 10-ml overnight cultures were used to prepare genomic DNA following the manual instructions for a bacterial DNA kit (Omega Bio-Tek, USA). The genomic DNA of GDIAS 001 was monitored using both 1% agarose gels and NanoDrop One (Thermo Fisher Scientific). Lastly, qualified DNA was used to construct a genomic DNA library with the SMRTbell template prep kit 1.0-SP v3 (Pacific Biosciences, USA), and then sequencing was conducted with PacBio RS II single-molecule real-time (SMRT) sequencing technology (Pacific Biosciences).

A total of 1.46 Gb of SMRT sequencing data (subreads) were obtained. These data were self-corrected, and preliminary assembly was done using Falcon v0.3+ with default settings (default parameters were used for all software unless otherwise specified) (8). The consensus sequence was corrected using Genomic Consensus software v2.3.3 and Spira v0.9.9.23. Lastly, the bacterial genome was circularized using Circlator v1.5.1 (9).

After filtering, 61,653 reads with an *N*_50_ value of 7,499 bp were assembled using the Hierarchical Genome Assembly Process (HGAP) pipeline of SMRT Analysis v2.3.0 (10) with genome coverage of 164×. The genome of P. pentosaceus GDIAS 001 consists of one circular chromosome of 1,831,351 bp with a 37.71% average DNA G+C content. Glimmer v3.0 (http://ccb.jhu.edu/software/glimmer/index.shtml) and Prodigal v2.6.3 (https://github.com/hyattpd/prodigal/releases/) were used to retrieve the related coding gene (11). tRNA genes were predicted using tRNAscan-SE v1.3.1 (12). rRNA genes were analyzed using RNAmmer v1.2 (13). Small nuclear RNAs (snRNAs) were predicted using BLAST against the Rfam database v10.0 (14). A total of 1,835 putative protein-coding genes and 71 RNA genes, including 15 rRNA (5 5S rRNAs, 5 16S rRNAs, and 5 23S rRNAs) and 56 tRNA genes, were predicted. The Virulence Factors Database (VFDB) (http://www.mgc.ac.cn/VFs) (15) was used to detect virulence factors, and 266 putative virulence factors were found in the genome of GDIAS 001. Proteins were predicted using SignaIP v4.1 (16), and 35 secretory proteins were presumed to contain signal peptides. The Antibiotic Resistance Genes Database v1.1 (http://ardb.cbcb.umd.edu/) (17) was used to evaluate the genomic sequences for antimicrobial resistance (AMR) genes, and three AMR genes (resistance to trimethoprim, elfamycin, and rifampin) were identified. The genomic sequence of GDAIS 001 was annotated by the NCBI PGAP (18) and deposited in the GenBank database.

### Data availability.

This whole-genome shotgun project, including raw reads of Pediococcus pentosaceus GDIAS 001, has been deposited at DDBJ/EMBL/GenBank under the accession number CP046938. The raw reads of Pediococcus pentosaceus GDIAS 001 have been deposited under SRA accession number SRR10716342.
